# Preparation and Anti-cancer Evaluation of Methotrexate-loaded Inositol-6 Phosphate Cross-linked Chitosan Nanoparticles on Breast Cancer

**DOI:** 10.34172/apb.43661

**Published:** 2025-03-08

**Authors:** Masoud Farshbaf, Nasrin Gobakhlou, Muhammad Sarfraz, Javid Shahbazi-Mojarrad, Mohammad Feyzizadeh, Hamed Hamishehkar, Parvin Zakeri-Milani, Hadi Valizadeh

**Affiliations:** ^1^Department of Medical Nanotechnology, Faculty of Advanced Medical Science, Tabriz University of Medical Science, Tabriz, Iran.; ^2^Student Research Committee, Faculty of Advanced Medical Sciences, Tabriz University of Medical Sciences, Tabriz, Iran.; ^3^College of Pharmacy, Al Ain University, Al Ain 64141, United Arab Emirates.; ^4^Drug Applied Research Center, Faculty of Pharmacy, Tabriz University of Medical Science, Tabriz, Iran.; ^5^Department of Pharmaceutics, Faculty of Pharmacy, Tabriz University of Medical Science, Tabriz, Iran.; ^6^Research Center of New Material and Green Chemistry, Khazar University, 41 Mehseti Street, AZ1096, Baku, Azerbaijan; ^7^Liver and Gastrointestinal Diseases Research Center, Faculty of Pharmacy, Tabriz University of Medical Science, Tabriz, Iran.

**Keywords:** Chitosan, Inositol hexaphosphate, Methotrexate, Breast cancer

## Abstract

**Purpose:**

Chitosan nanoparticles (CNs) have directed considerable research efforts towards developing biocompatible, biodegradable, inexpensive and efficient particulate drug delivery systems.

**Methods:**

In the present investigation, we utilized green and safe inositol hexaphosphate (InsP6) as a physical cross-linker to obtain CNs (^InsP6^CNs) and compared their size, zeta potential and cell uptake ability with the CNs cross-linked with tripolyphosphate (TPP) as a commonly used cross-linker (^TPP^CNs). Methotrexate (MTX) as the model drug was physically incorporated within the both types of CNs (^InsP6^CNs_MTX_ and ^TPP^CNs_MTX_) and their time-dependent anti-cancer behavior was evaluated on MCF-7 cell line.

**Results:**

Compared to ^TPP^CNs, ^InsP6^CNs were bigger in hydrodynamic diameter and showed far different zeta potential value. The MTX encapsulation efficiency was much higher for ^InsP6^CNs_MTX_ than that of ^TPP^CNs_MTX_. ^InsP6^CNs and ^TPP^CNs showed similar *in vitro* cell uptake behavior, examined on MCF-7 cell line. Furthermore, after 24 h, ^InsP6^CNs_MTX_ had the most *in vitro* antitumor effect on the MCF-7 cells, compared to free MTX and ^TPP^CNs_MTX_.

**Conclusion:**

Consequently, InsP6 can be presented as an accessible and cost-effective member of physical cross-linkers to prepare efficient CNs as drug delivery systems.

## Introduction

**Figure 1:**
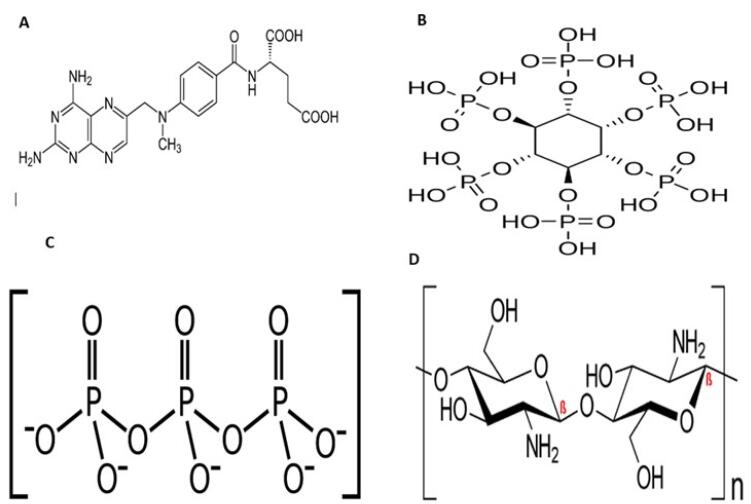
Chemical structures of MTX (A), InsP6 (B), TPP (C), and Chitosan (D)

 Chitin is one the most abundant polysaccharides in nature consisting β-1,4-linked glucosamine as its sugar backbone with a high degree of *N*-acetylation.^1^ Chitosan, the main derivative of chitin, is a natural biodegradable cationic polymer with a random distribution of *N*-acetyl glucosamine and D-glucosamine in its backbone and commonly obtained by alkaline deacetylation.^2^ Due to its unique biological properties including nontoxicity, biocompatibility, biodegradability and mucoadhesive behavior, chitosan has attracted much of attention upon using in different fields of biomedical applications such as drug and gene delivery systems and biomedical engineering.^3,4^ Having the pKa around 6.5, chitosan is considered as a pH-sensitive polymer, by which at the pH values beneath this pKa, the amine groups of chitosan get protonated, forming polyelectrolyte with high charge density. On the other hand, at pH values near neutral (7-7.4), those amine groups become deprotonated and decrease the charge density of polymer. This behavior also influences the soluble-insoluble transition of chitosan. Among the many of techniques to obtain chitosan nanoparticles (CNs), emulsion cross-linking has been commonly employed in recent years.^5^ Cross-linking agents possess at least two reactive sites by which they can form bridges between the polymer chains. There are two different types of cross-linkers including chemical and physical. Through intra- and intermolecular covalent bindings, the reactive site of chemical cross-linkers like genipin, cinnamaldehyde and glutaraldehyde react with the amine groups of chitosan and form a complex, irreversible and unresponsive net of polymer and consequently rigid CNs.^6,7^ On the other hand, physical cross-linkers are mostly negative charged ions which form electrostatic interactions with the cationic amine groups of chitosan. This method is facile and the obtained nanoparticles have massive hydrophilic capacity within, presenting pH-responsive ability and are more biocompatible than the previous ones.^8^ Tripolyphosphate (TPP) is the most employed physical cross-linker for the CNs preparation,^9^ though it is a chemical agent, and besides being expensive to provide, it may cause cytotoxic effects upon using in the body. Thus, it is worth introducing a safer, cheaper and more accessible alternative. Inositol hexaphosphate (InsP6) is a vitamin-like naturally occurring polyphosphorylated carbohydrate mostly found in oil seeds, nuts, soybean and animals. InsP6 has been proved with anti-cancer effect on prostate,^10^ colorectal^11^ and breast cancers.^12^ Considering the chemical structure of InsP6, as a polyphosphate species, it is capable of forming ionic interactions with chitosan and acting as a green and accessible physical cross-linker with therapeutic action (Figure 1).^13^ In this study, we use InsP6 as a green cross-linker to obtain CNs (^InsP6^CNs) loaded with methotrexate (MTX) (^InsP6^CNs_MTX_) as an anti-cancer model drug. For comparison, the CNs cross-linked with TPP (^TPP^CNs) loaded with the same drug (^TPP^CNs_MTX_) was also prepared.

## Materials and Methods

###  Materials

 Chitosan (medium molecular weight), MTX, fluorescein isothiocyanate (FITC), TPP and InsP6 were purchased from Sigma-Aldrich (St. Louis, MO, USA). RPMI1640 medium and fetal bovine serum (FBS) were provided by Gibco® (Grand Island, NY, USA). 3-(4, 5-dimethylthiazol-2-yl)-2, 5 diphenyltetrazolium bromide (MTT) was purchased from CARL ROTH (Roth, Karlsruhe, Germany). MCF-7 Cell Line was obtained from Pasteur Institute (Tehran, Iran). All the other organic reagents were of analytical grade and purchased from Merck (Darmstadt, Germany).

### *Synthesis of* ^InsP6^CNs

^InsP6^CNs were synthesized by a facile method using InsP6 as a green and safe cross-linker for chitosan at an acidic environment. Briefly, 105 mg of chitosan was added to 35 mL of acetic acid (1%) in a round-bottom flask. After fine dispersion of chitosan in the solution, 14 mL of a mixture with concentration of 1 mg mL^-1^ of InsP6 in deionized (DI) water was added drop-wise to the above solution under stirring (600 rpm) at 65 °C for 30 minutes. Once the solution became almost opaque, the stirring speed was decreased to 400 rpm for 1 min and the nanoparticles were settled down. Finally, the supernatant was collected and the obtained ^InsP6^CNs were washed using DI water and then re-dispersed in fresh DI water using the Ultrasonic Cleaner (Otto, Italy).

####  Synthesis of ^TPP^CNs

 The procedure for preparation of ^TPP^CNs was similar to that of ^InsP6^CNs using the same concentrations and with only difference in the type of cross-linker and reaction temperature which was carried out by using TPP and at ambient temperature, respectively.

###  Synthesis of ^InsP6^CNs_MTX_ and ^TPP^CNs_MTX_

 In order to physically encapsulate the MTX within the ^InsP6^CNs and ^TPP^CNs, a 2.2 mg mL^-1^ solution of MTX was obtained by dissolving it in the cross-linker solution (InsP6 or TPP) prior to addition to the chitosan solution. The rest of the protocol was same as for the synthesis of blank ^InsP6^CNs or ^TPP^CNs. After synthesis of ^InsP6^CNs_MTX_ and ^TPP^CNs_MTX, _the unloaded drug was separated by centrifugation (Hettich, Germany) for 10 min at 5000 rpm. In order to calculate the encapsulation efficiency (EE) of MTX in both ^InsP6^CNs_MTX_ and ^TPP^CNs_MTX_, the collected supernatants were analyzed using ultraviolet-visible (UV-Vis) spectrophotometer (Shimadzu, Japan) at λ_max_ = 306 nm and the calculations were carried out according to the equation 1:


Eq. 1 $$EE\;of\;MTX\%=\frac{MTX\;added\left(mg\right)-MTX\;in\;supernatant\left(mg\right)}{MTX\;added\;\left(mg\right)}⊗100$$

###  Synthesis of fluorescence CNs

 In order to prepare FITC-tagged CNs (^InsP6^CNs_FITC_ and ^TPP^CNs_FITC_), 10 mg FITC (0.71 mg mL^-1^) was added to the cross-linker solution (InsP6 or TPP) prior to addition to the chitosan solution. The rest of the protocol was same as for the synthesis of blank ^InsP6^CNs or ^TPP^CNs, and carried out in a dark room to prevent photo-leaching.

###  Characterization techniques

 The mean hydrodynamic diameter, polydispersity index (PDI), and zeta potential of ^InsP6^CNs and ^TPP^CNs were assessed using Nano S zetasizer (Malvern Instruments, Malvern, UK). The samples were diluted with DI water and the mean hydrodynamic diameter and zeta potential were measured in three replicates at pH = 7.4 and 25 °C.

###  Cell culture and in vitro cell viability assay 

 Breast cancer MCF-7 cell line was cultured according to the ref^14^ and used to investigate the *in vitro* antitumor effect of ^InsP6^CNs_MTX_ and ^TPP^CNs_MTX_ and cellular uptake of ^InsP6^CNs_FITC_ and ^TPP^CNs_FITC_. Briefly, cells were cultured in Gibco® RPMI1640 containing 10% (v/v) FBS, 100 U mL^-1^ penicillin G, and 100 µg mL^-1^ streptomycin and incubated at 37 °C in an atmosphere of 5% CO_2_ and 95% airfor 24 h. After reaching to proper population and their detachment with 0.25% trypsin in phosphate buffer saline (PBS), cells were seeded in 96-well plates with cell density of 1.0 × 10^4^ cells/well and allowed to grow for 24 h with the same temperature and atmosphere. Then, in order to perform time-dependent viability study, cells were treated with equivalent concentrations of chitosan, TPP, InsP6, MTX,^TPP^CNs,^InsP6^CNs, ^InsP6^CNs_MTX_ and ^TPP^CNs_MTX_. Equivalent volume of fresh mediumwasadded to control group. After incubation for 24 h the MTT assay was carried out in triplicates. To this end, the old media was removed and 100 µL of fresh medium with 10 µL of a 5 mg mL^-1^ MTT in PBS was added to each well and after covering with aluminum foil, incubated for 4 h. The old medium was replaced with 100 µL DMSO in order to dissolve the formazan crystals. After shaking for 10 min, an ELISA plate reader(Bio-Tek Instruments, USA) was used to measure the absorbance of each well at 570 nm with a background correction at 630 nm.

###  Study of in vitro cellular uptake

 The MCF-7 cells with density of 5 × 10^5^ cells/well were seeded into 6-well plate and incubated for 20 h. Then, the culture medium was replaced with 100 µl fresh medium (with no antibiotic) containing proper amount of ^InsP6^CNs_FITC_ or ^TPP^CNs_FITC_ (2 wells for each sample) and incubation was continued at 37 °C. After 90 min, the fluorescent intensity and quantitative analysis were evaluated by flow cytometry (FACSCalibur, Becton Dickinson, San Jose, CA, USA).

###  Statistical analysis

 Data analysis was conducted using GraphPad Prism version 6. Data were represented as mean ± standard deviation (SD). One way analysis of variance (ANOVA) was used to perform comparisons between groups. *P* value < 0.05 was considered statistically significant.

## Results and Discussion

###  Synthesis of nanoparticles

 In this study, we used a facile method in which InsP6 acted as a naturally occurring and non-harmful physical cross-linker to prepare CNs in an acidic medium. In order to compare the results, we also synthesized CNs with the commonly used synthetic cross-linker, TPP. When the cross-linker solution (InsP6 or TPP) was being added dropwise, the formation of either ^InsP6^CNs or ^TPP^CNs was obvious by gradually turning the color of solution to opaque. FITC was used as a fluorescence dye to tag the ^InsP6^CNs and ^TPP^CNs and to evaluate their *in vitro* cell uptake behavior. FITC was physically encapsulated within the nanoparticles via the electrostatic interaction of its hydroxyl groups with the amine groups of CNs resulting in a mild colorful suspension. Similarly, MTX was trapped into the ^InsP6^CNs and ^TPP^CNs with proposed interactions of its pteridine ring by negative phosphate groups. EE of MTX in ^InsP6^CNs_MTX_ and ^TPP^CNs_MTX_ was calculated to be 32.5% and 8.2%, indicating the superior ability of ^InsP6^CNs to encapsulate the MTX. This could be due to the fact that, the encapsulation of MTX was basically dependent on the electrostatic interactions of its cationic end groups with the anionic parts of cross-linker and chitosan. As InsP6 has higher negative net charge than its counterpart, TPP, the higher MTX EE for ^InsP6^CNs_MTX _was expected.^15^

###  Size and charge of ^InsP6^CNs and ^TPP^CNs

**Figure 2:**
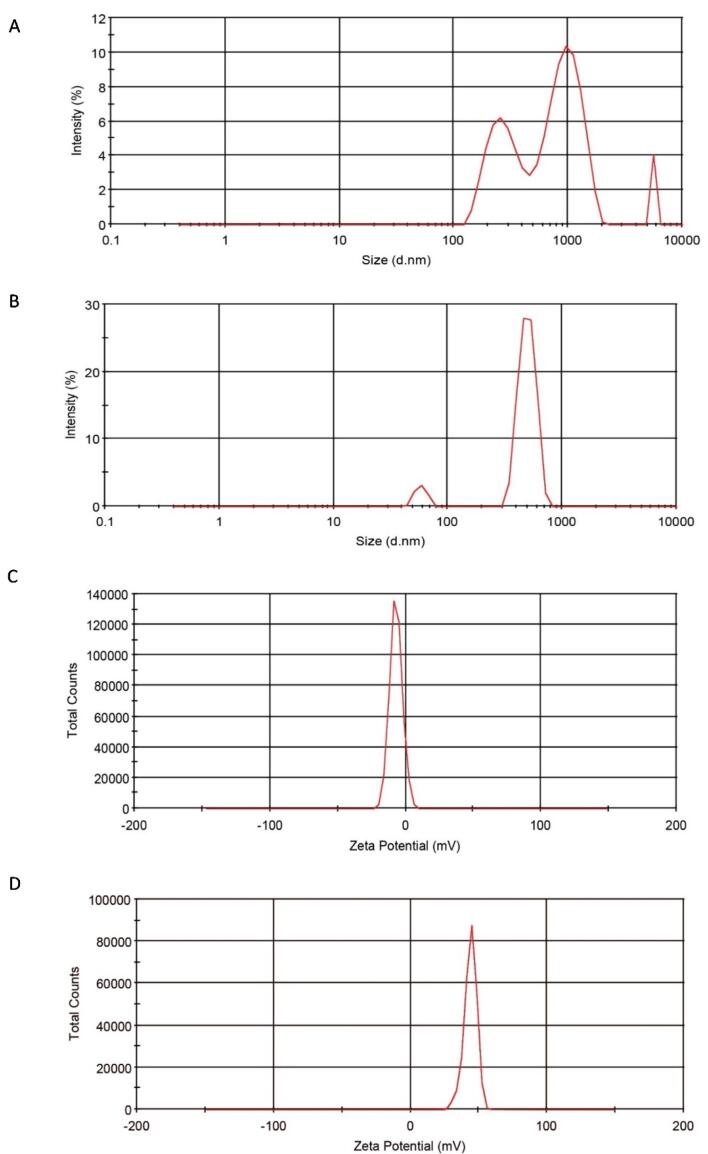
Size distribution of InsP6CNs and TPPCNs (A, B), and zeta potential of InsP6CNs and TPPCNs (C, D)

 The hydrodynamic diameter of ^InsP6^CNs and ^TPP^CNs were measured in DI water using dynamic light scattering (DLS) instrument and based on the light scattering profile of nanoparticles. The mean size of ^InsP6^CNs and ^TPP^CNs were measured to be 918.5 ± 103.6 nm and 463.3 ± 21 nm, respectively. CNs cross-linked with InsP6 were bigger in size than those of with TPP, which could be possibly due to the different molecular structure and reactive site of InsP6 and its higher negative net charge compared to TPP. Furthermore, ^TPP^CNs were relatively mono-disperse with narrow range of size distribution (Figure 2A) compared to poly-disperse behavior of ^InsP6^CNs having wide range of size distribution (Figure 2B). Thus, it is better to use TPP as cross-linker, if smaller CNs are required for a specific purpose. Of course, smaller ^InsP6^CNs may also be obtained by applying diverse changes in synthesis procedure like using different concentrations of chitosan and InsP6 and different solvents, and also benefiting from some of the top-down techniques such as high pressure homogenizer (HPH).^16^ The zeta potential values for ^InsP6^CNs and ^TPP^CNs were -6.97 ± 2.36 mV and + 43.9 ± 12.51 mV, respectively (Figure 2C, 2D), which could be due to the higher negative charge density of InsP6 compared to that of TPP. The higher cationic surface charge of ^TPP^CNs resulted in electrostatic repulsion between the nanoparticles and kept them dispersed in DI water, while there was relatively lower stability shown with ^InsP6^CNs against agglomeration. However, in drug delivery systems, the immune responses are insignificant to negatively charged nanoparticles and they are reported to be less toxic than highly destructive cationic ones.^17^

###  In vitro cell viability assay

**Figure 3:**
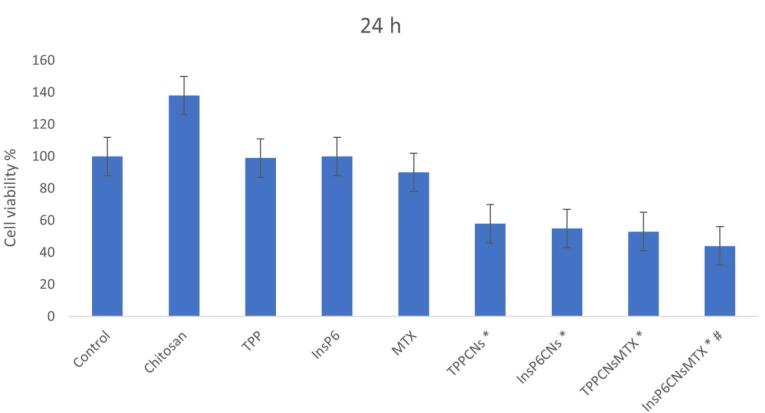
Anti-proliferation effect of different formulations after 24 h against MCF-7 cells; n = 3; * *p* < 0.01 compared to control group, # *P* < 0.05 compared to MTX group

 The therapeutic efficiency of ^InsP6^CNs_MTX_ and ^TPP^CNs_MTX_were studied compared to each other along with TPP, InsP6, MTX, chitosan, ^InsP6^CNs, and ^TPP^CNs using MTT assay on MCF-7 cell line after 24 h of incubation. As shown in Figure 3, after 24 hours, ^InsP6^CNs_MTX_ exhibited highest time-dependent cell cytotoxicity compared to other groups especially to those which were treated with ^TPP^CNs_MTX_ and free MTX, that may be due to the combination of higher MTX loading of ^InsP6^CNs_MTX_ and anti-cancer behavior of InsP6, which was shown in cells treated with free InsP6. Both of the blank^InsP6^CNs and ^TPP^CNs showed cytotoxic behavior, mainly because of above-mentioned anti-cancer behavior of InsP6 involved within the^InsP6^CNs and high cationic charge of ^TPP^CNs which disrupted the cell membrane and resulted in cell death. Interestingly, free chitosan enhanced the cell proliferation, indicating the fact that the cells might use chitosan as a power supply.

###  In vitro cellular uptake

**Figure 4:**
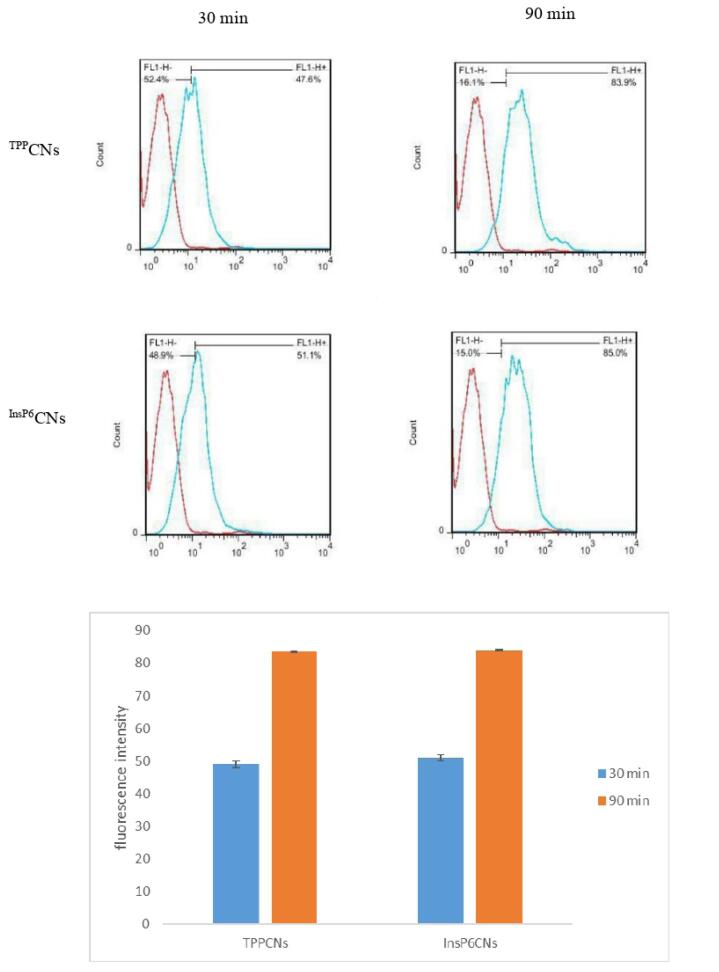
Cellular uptake of FITC-tagged InsP6CNs and TPPCNs on MCF-7 cells after 30 and 90 min

 The *in vitro* cell uptake ability of ^InsP6^CNs_FITC_ and ^TPP^CNs_FITC_on MCF-7 cells was evaluated quantitatively after 30 and 90 minutes using flow cytometry. Based on florescence intensity, both samples showed similar cell uptake behavior (Figure 4) and as expected, by increasing the incubation time to 90 min, the cell uptake was enhanced for both ^InsP6^CNs_FITC_ and ^TPP^CNs_FITC_. Moreover, as MTX has similar structure to folic acid, it can act as a potential ligand for the overexpressed folate receptors onto the cancer cells allowing the ^InsP6^CNs_MTX_ and ^TPP^CNs_MTX_ into the cells through receptor-mediated endocytosis.^14^

## Conclusion

 In the current study, we presented InsP6 as a member of physical cross-linkers for CNs preparation. The synthesis procedure was quite similar for both of ^InsP6^CNs and ^TPP^CNs. Though, ^InsP6^CNs were bigger in size, but they possessed negative zeta potential compared to highly cationic ^TPP^CNs, which can reduce the following charge-related cytotoxicity. That is why the MTT assay emphasized on the cytotoxic behavior of blank ^TPP^CNs on MCF-7 cells. Due to enhanced electrostatic interactions and better physical entrapment of MTX within the carrier, ^InsP6^CNs_MTX _showed higher encapsulation efficiency than ^TPP^CNs_MTX_. ^InsP6^CNs_MTX_ showed excellent time-dependent *in vitro* antitumor behavior compared to ^TPP^CNs_MTX_ and free MTX. Moreover, both ^InsP6^CNs and ^TPP^CNs presented similar *in vitro* cellular uptake capability towards MCF-7 cells. In conclusion, InsP6 can be employed as a promising physical cross-linker to attain efficient CNs for drug delivery applications.

## Competing Interests

 The authors declare that they have no competing interests. The authors alone are responsible for the content and writing of this article.

## Ethical Approval

 This study was approved by research ethical committee of Tabriz university of medical sciences.
